# Retraction Note: LncRNA HOTAIRM1, miR-433-5p and PIK3CD function as a ceRNA network to exacerbate the development of PCOS

**DOI:** 10.1186/s13048-026-01983-5

**Published:** 2026-01-26

**Authors:** Hongmin Guo, Ting Li, Xinhui Sun

**Affiliations:** 1 Department of Reproductive Medicine, Liaocheng People’s Hospital, NO.67, Dongchang West Road, Liaocheng City, Shandong Province 252000 P. R. China; 2 College of Bioengineering, Wuhu Institute of Technology, NO.201, Wenjin West Road, Wuhu City, Anhui Province 241103 P. R. China

**Retraction Note: J Ovarian Res 14, 19 (2021)**

**https://doi.org/10.1186/s13048-020-00742-4**

The Editors-in-Chief have this article because it contains data that overlaps with data from the following article [1], published by *Cancer Management and Research*. In addition, an investigation conducted after the publication determined that the two articles were under consideration by their respective journals at the same time. The authors did not provide an explanation for this data overlap. The Editors-in-Chief therefore no longer have confidence in the results and conclusions presented in this article.

The authors disagree with this retraction.
